# Ganglion Cell and Displaced Amacrine Cell Density Distribution in the Retina of the Howler Monkey (*Alouatta caraya*)

**DOI:** 10.1371/journal.pone.0115291

**Published:** 2014-12-29

**Authors:** José Augusto Pereira Carneiro Muniz, Luana Modesto de Athaide, Bruno Duarte Gomes, Barbara L. Finlay, Luiz Carlos de Lima Silveira

**Affiliations:** 1 Centro Nacional de Primatas, Ananindeua, Pará, Brazil; 2 Instituto de Tecnologia, Universidade Federal do Pará, Belém, Pará, Brazil; 3 Instituto de Ciências Biológicas, Universidade Federal do Pará, Belém, Brazil; 4 Department of Psychology, Cornell University, Ithaca, New York, United States of America; 5 Núcleo de Medicina Tropical, Universidade Federal do Pará, Belém, Pará, Brazil; Federal University of Rio de Janeiro, Brazil

## Abstract

Unlike all other New World (platyrrine) monkeys, both male and female howler monkeys (*Alouatta sp.*) are obligatory trichromats. In all other platyrrines, only females can be trichromats, while males are always dichromats, as determined by multiple behavioral, electrophysiological, and genetic studies. In addition to obligatory trichromacy, *Alouatta* has an unusual fovea, with substantially higher peak cone density in the foveal pit than every other diurnal anthropoid monkey (both platyrrhines and catarrhines) and great ape yet examined, including humans. In addition to documenting the general organization of the retinal ganglion cell layer in *Alouatta*, the distribution of cones is compared to retinal ganglion cells, to explore possible relationships between their atypical trichromacy and foveal specialization. The number and distribution of retinal ganglion cells and displaced amacrine cells were determined in six flat-mounted retinas from five *Alouatta caraya*. Ganglion cell density peaked at 0.5 mm between the fovea and optic nerve head, reaching 40,700–45,200 cells/mm^2^. Displaced amacrine cell density distribution peaked between 0.5–1.75 mm from the fovea, reaching mean values between 2,050–3,100 cells/mm^2^. The mean number of ganglion cells was 1,133,000±79,000 cells and the mean number of displaced amacrine cells was 537,000±61,800 cells, in retinas of mean area 641±62 mm^2^. Ganglion cell and displaced amacrine cell density distribution in the *Alouatta* retina was consistent with that observed among several species of diurnal Anthropoidea, both platyrrhines and catarrhines. The principal alteration in the *Alouatta* retina appears not to be in the number of any retinal cell class, but rather a marked gradient in cone density within the fovea, which could potentially support high chromatic acuity in a restricted central region.

## Introduction

Platyrrhines (New World monkeys) differ from catarrhines (Old World monkeys, gibbons, apes, and humans) in the variety of color vision phenotypes they exhibit. In catarrhine species, Old World monkeys and great apes, both males and females are trichromats, because in both males and females, three different genes (two in the X-chromosome and one in chromosome 7) code three different opsins. These opsins show the appropriate separation of light absorption peaks, and each opsin is expressed in a separate cone class [1]–[3]. In addition, the presence of single-cone midget bipolar cells [4]–[5] and retinal post-receptoral neural circuits provide color opponent mechanisms to produce full trichromacy in all individuals [6]–[11].

In most platyrrhine species, by contrast, only a proportion of females are trichromats (regular or anomalous). The remaining females and all males are dichromats because there are only two coding genes for opsins, one in the X-chromosome and another in the chromosome 7. In males, with only one X-chromosome and homozygous females, the result is dichromacy, while in heterozygous females gene polymorphism permits trichromacy [12]–[13]. The nocturnal owl monkey, *Aotu*s *sp.*, is an exception, a monochromat with a single cone class [14]–[15]. Like catarrhines, the platyrrhine retina has single-cone midget bipolar cells [16]–[17] and the necessary post-receptoral mechanisms for trichromacy [18]–[20]. Given this common retinal architecture, and the presence of three separate opsins, both male and female *Alouatta* are regular trichromats and their color vision seems to be very similar to that of catarrhines [21]–[22].

Even though their color vision phenotypes are quite different, catarrhines and platyrrhines are quite similar in most aspects of their retinal anatomy. They have similar classes of horizontal cells [23], bipolar cells [16]–[17], and ganglion cells [20], [24]–[32]. The absolute foveal area in platyrrhines and catarrhines is conserved across a number of species [33]. Diurnal catarrhines and platyrrhines with similar retinal area have similar density distributions of cones, rods, and ganglion cells [33]–[35]. Comparisons between a large group of diurnal catarrhines and platyrrhines showed a regular scaling of all retinal neurons in diurnal species with respect to both eye diameter and brain volume [35]. This lawful scaling of rods, cones, and retinal ganglion cell number was hypothesized to result from a conserved sequence of cell generation that defends retinal acuity and sensitivity over a large range of eye sizes [35]–[36].

There are only few studies of *Alouatta* retinal anatomy. Franco and colleagues were able to estimate the cone density for both retinas of one *Alouatta* and found that they had an extremely high cone density in the foveal pit, about 429,000 cones/mm^2^ and 357,000 cones/mm^2^, respectively, accompanied by a comparably reduced cone diameter [33]. This density is higher than any other primate ever described, including humans with 324,000 cones/mm^2^ [37]. It is not known whether the density of retinal ganglion cells matches the atypical high density of cones faithfully, preserving typical convergence ratios, or if there is greater apparent convergence of cones to ganglion cells, as determined from their ratio, compared to other diurnal monkeys. In the present work, the density distributions of ganglion cells and displaced amacrine cells were determined in six retinas from five *Alouatta caraya* (central counts were performed in five retinas). The results show that the neurons of the ganglion cell layer are distributed following roughly the same pattern observed in other platyrrhines and also in catarrhines with minor specific differences. The overall ratio of cones to retinal ganglion cells for the entire fovea and foveolar region is as expected for other diurnal primates, so an acuity benefit from the region of high maximum density in *Alouatta* would have to be compensated by an acuity decrement in neighboring regions.

## Materials and Methods

### Ethics statement

All animal experiments were carried out in accordance with the National Institute of Health Guide for the Care and Use of Laboratory Animals (NIH Publications No. 80–23, revised 1996), and were approved by the Ethical Research Committee for Animal Experiments of the Institute of Biological Sciences, Federal University of Pará (#MED004/2008).

### Animals

Five adults, male *Alouatta caraya* were obtained from the breeding colony of the Centro Nacional de Primatas – CENP (Ananindeua, Pará, Brazil). The animals were bred and kept in the CENP facilities until the day the experiment started. There, they were kept in housing conditions permitting social interaction with their conspecifics and submitted to an appropriate feeding regimen and drinking water *at libitum*. Animal housing conditions, feeding regimen, and health were supervised by the CENP veterinarian staff. The animals were kept in environmental enriched cages as recommended by CENP primatologists.

Each animal room measured 2.50 m×4.00 m×2.25 m, 10 m^2^ of total area. Each room pair housed 1 male and 5–6 female *Alouatta*. Houses and rooms were cleaned daily. They were washed with jets of water containing 10% hypochlorite. Animals were moved from one of the paired rooms to the other during cleaning. Clean water was provided for primates in 500 ml animal drinking bottles, two bottles per room. Bottles were refilled 3 times per day. Primate diet consisted of the following items: a) food pellets provided in plates, ad libitum; b) fruits, vegetables, once a day; cooked eggs, twice a week; diluted milk, twice a week. In addition, *Alouatta* were provided, daily with leaves from Embauba tree, which are part of the natural diet of these Amazonian primates. All animals housed in the CENP received continuous veterinary care which followed the following protocol: inspection in the first hour every day to observe each animal and to inspect the feeding plates and drinking bottles; thereafter regular inspection until the evening. There are also facilities for primate anesthesia and surgery. The facilities allow primate full veterinary assistance, including X-ray, ultrasound, and complete blood, urine, and stool clinical laboratory examination and testing. Each room is enriched with trunks, branches, and ropes to facilitate primate exercising and escape during disputes and force display. Platforms are available for resting and access for feeding.

On the day of experiment, the animals were sedated with an intramuscular injection of ketamine (ca 20 mg kg^−1^) and then transferred to the Biological Sciences Institute of the Federal University of Para. The animal's electrocardiogram (ECG) was then recorded with thoracic surface electrodes, amplifier, and oscilloscope (Nihon Kohden Co, Tokyo, Japan). Then, the animals were euthanized with an overdose of barbiturate, Thionembutal (Abbott, Abbott Park, Illinois, USA), 35 mg/kg or higher. Death was assessed by cessation of electrocardiogram ECG activity.

Six retinas were studied from the five animals. The right retinas of four animals were used for experiments not connected with this work while the left retinas were used in this work (AC 01L, AC 02L, AC 03L, and AC 04L). The two retinas of the fifth animal were used in this work only (AC 05L and AC 05R).

### Retinal whole mounts

Animals were initially deeply anesthetized with the following intravenous anesthetics mixture: Levomepromazine (Neozine; Sanofi-Avensis, São Paulo, São Paulo, Brazil) 0.2–0.4 mg/kg; Midazolan (Dormicum; Roche, São Paulo, São Paulo, Brazil), 0.2–0.4 mg/kg; Ketamine Chloridrate (Vetanarcol; König, São Paulo, São Paulo, Brazil), 10–15 mg/kg. Just before perfusion they received Sodium Heparine 5,000 IU/ml (Roche), 1 ml i.v., to prevent blood coagulation followed by an intravenous lethal dose of Thionembutal (Abbott, Abbott Park, Illinois, USA), 35 mg/kg or higher. ECG was continuously monitored to ensure adequate depth of anesthesia and analgesia. Death was assessed by cessation of ECG activity. They were then transcardially perfused with 0.9% phosphate buffered saline solution (PBS, pH 7.2), 500 ml, followed by 10% formaldehyde in PBS, 2000 ml. The eyes were removed, the retinas were dissected, mounted on a gelatinized slide with the ganglion cells uppermost, exposed to formaldehyde vapor 60°C for 2–3 h, then stained by the method of Nissl. Briefly, the retinas were rehydrated, stained with 0.5% cresyl violet (Merck, Darmstadt, Germany) 50–60°C under microscopic control, dehydrated in graded alcohols, cleared in xylene, and coverslipped.

### Measurements of optic disk size, distance from the optic disk to the fovea, and retinal area

Measurements of retinal area were performed just after retinal dissection and in the end of all histological procedures. The results were compared in order to estimate retinal shrinkage. Retinal contours were drawn using a photographic enlarger at ×5 magnification and digitized using a HP Photosmart C5180 multifunctional equipment and the HP Scan and Camera Wizard software (Hewlett Packard, Palo Alto, California, USA). Retinal area was then measured using the AutoCAD 2007 software (Autodesk, San Rafael, California, USA).

The positions of the fovea and optic disk center were also marked in the retinal drawings and their distance measured before and after histological procedure. The results were used to estimate if any shrinkage occurred in the central regions of the retina or were restricted to retinal borders and cuts made to obtain the flat mount. In addition, the vertical and horizontal lengths of the optic disk were measured on the microscope using a calibrated eyepiece.

### Ganglion cell and displaced amacrine cell counts

The retinal whole mounts were used to count ganglion cells and displaced amacrine cells along 12 radial directions, at 30° intervals, extending from the fovea towards retinal borders. The 0° and 180° directions hemisected the retina in the nasotemporal dimension passing through the fovea and optic disk while the 90° and 270° directions hemisected the retina in the dorsoventral dimension and passed through the fovea. These two lines were perpendicular to each other at the fovea and were called, in this work, horizontal and vertical meridians, respectively. Several works performed in primates in our laboratories and other laboratories used these two meridians as references, making possible to compare their results [34], [38].

Counts were performed under microscopic observation, using an ×100 oil immersion objective and ×10 eyepieces. The fovea was taken as the coordinate origin and counts were made at regular intervals from the fovea towards retinal borders. To account for the rapid cell density change in the first millimeters of distance from the fovea, counts were made at 0.25 mm intervals in the first 3 mm and then at 1 mm intervals up to the retinal borders. In addition, the counting area was 1,537 µm^2^ in the first 1.5 mm from the fovea, then increased to 9,604 µm^2^ from 1.75 up to 5 mm, and then increased again to 38,416 µm^2^ from 6 mm up to the retinal periphery. Results were recorded as cells/mm^2^ on enlarged maps of the flat mounted retina, at ×10 magnification for the entire retina and ×40 magnification for the central 3 mm.

### Isodensity maps and estimate of the total number of ganglion cells and displaced amacrine cells

From the density values of ganglion cells and displaced amacrine cells measured at each retinal location, enlarged isodensity maps were drawn to illustrate the density topography for such cells of the *Alouatta* retina. The isodensity contours were plotted linking points with density corresponding to the isodensity contour and points located between densities higher and lower than that corresponding to the isodensity contours. The total number of ganglion cells and displaced amacrine cells were estimated by measuring the area between two isodensity contours, multiplying the area times the mean density value of the two isodensity contours, and adding all the resulting figures.

### Statistics analysis

Statistical analysis of the results was performed using software BioEstat 5.0 [39] and Microsoft Excel 2007 (Microsoft Corporation, Redmond, Washington, USA).

## Results

### *Alouatta* retinal ganglion cell layer

Six retinal whole mounts were successfully stained by the method of Nissl using cresyl violet as stain. Table 1 lists the optic disk dimensions and the distance from the center of the optic disk to the foveal pit measured in this collection of retinas. The mean values were 1.38±0.07 mm and 1.23±0.09 mm for the vertical and horizontal diameters of the optic disk, and 3.18±0.18 mm for the optic disk – foveal pit distance.

**Table 1:** Dimensions of the howler monkey optic disk (papila) along the dorsoventral (DV) and nasotemporal (NT) dimensions (N = 5) and the distance from the center of the optic disk to the foveal pit (N = 6).

Retina	Papila (DV) (mm)	Papila (NT) (mm)	Papila – Fovea (mm)
AC 01LM	1.35	1.18	3.43
AC 02LM	1.35	1.27	3.19
AC 03LM	-	-	2.89
AC 04LM	1.46	1.23	3.28
AC 05LM	1.35	1.35	3.23
AC 05RM	1.27	1.10	3.06
Mean	1.38	1.23	3.18
Standard deviation	0.07	0.09	0.18

AC, *Alouatta caraya*. R, right retina. L, left retina. M, retina from a male howler monkey.

**Figure 1:**
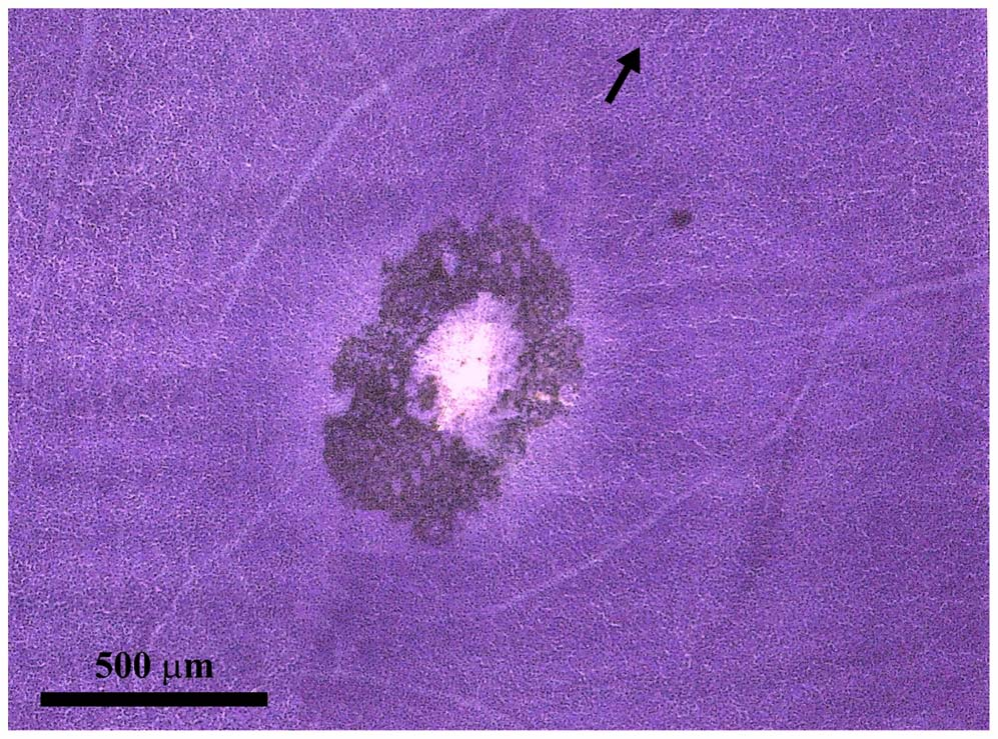
Foveal region of an *Alouatta* retina. Retinal flat mount AC 02 Left Male stained by the method of Nissl using cresyl violet as stain. Some retinal pigment remained attached to the region surrounding the foveal pit. The arrow points towards the location of the optic nerve head, which is out of the field. The retinal raphé is located on the opposite side in relation to the fovea and is indicated by the convergence of retinal vessels.

Fig. 1 shows an *Alouatta* retina mounted with the ganglion cell layer facing the observer and stained by the method of Nissl. As in other platyrrhines and catarrhines, cell bodies of ganglion cells and displaced amacrine cells were radially displaced from the foveal pit and then packed in the several strata of the foveal slope. In the foveal slope, cell body density was very high, decreasing towards retinal periphery in all directions.

**Figure 2:**
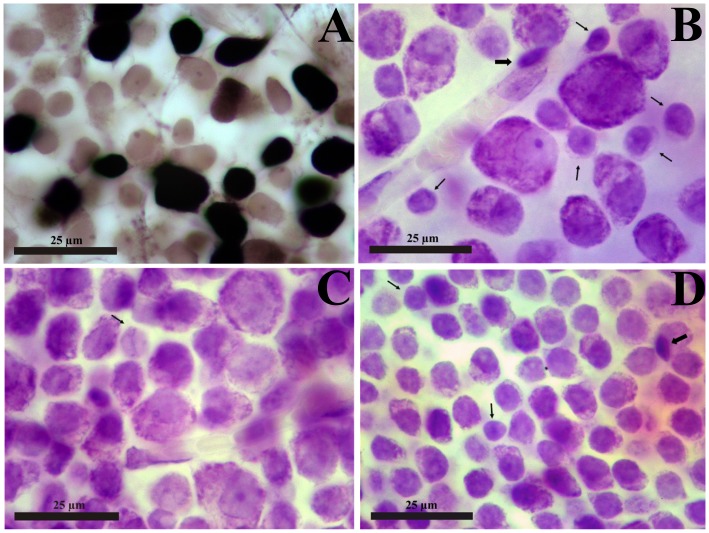
Retinal ganglion cell layer of New World monkeys. (**A**) Retinal flat mount of a *Cebus* monkey (right retina, male animal), focus on the ganglion cell layer, 1.8 mm temporal to the fovea. Cells were labeled by Biocytin retrograde transport after deposits of the neurotracer in the optic nerve, 2 mm behind the eyeball, followed by 24 hs survival time [40]. The retina was dissected free from the other retinal layers and the retinal pigment epithelium, incubated in Vectastain ABC System solution, reacted for peroxidase histochemistry using diaminobenzidine as chromophore, and exposed to osmium tetroxide to intensify the reaction product. All retrograde labeled cells are retinal ganglion cells showed in the picture as black stained cell bodies (axons and dendrites are also labeled but are largely out of focus in this photomicrograph). Cells that were not retrograde labeled are the light brown cell bodies in the picture and were stained by the weak unspecific diaminobenzidine reaction intensified by the osmium tetroxide exposure: they comprised some ganglion cells, displaced amacrine cells, microglia, astrocytes, and endothelial cells. This procedure was used to consolidate the criteria for identify and counting ganglion cells and displaced amacrine cells in New World monkeys (see text for details). (**B–D**) *Alouatta* retinal flat mount (AC 04 Left Male), stained by the method of Nissl using cresyl violet as stain. The retinal ganglion cell layer is shown at three increasing eccentricities: 5 mm (**B**), 2 mm (**C**), and 1 mm (**D**) temporal to the fovea. Ganglion cells comprise a heterogeneous population of neurons readily distinguishable from other neurons (thin arrows), glial cells (thick arrows), and endothelial cells. The non-ganglion cell neurons of the retinal ganglion cell layer comprise displaced amacrine cells and displaced horizontal cells, the later relatively rare in comparison with the former [42]–[44]. The glial cells comprise microglia (thick arrow in **B**) and astrocytes (thick arrow in **D**), which are generally located internally (microglia) or externally (astrocytes and microglia) to the retinal ganglion cells. The endothelial cells can be distinguished by forming the blood vessel walls (**B** and **C**). In despite the large increase in ganglion cell density and decrease in the sizes of ganglion cell bodies, the criteria to distinguish between cell classes of the retinal ganglion cell layer can be applied throughout the retinal flat mount, including regions close to the fovea. In the foveal slope (**D**) the ganglion cells are stacked in 4–7 cell layers and careful focus through the ganglion cell layer thickness is necessary to perform precise neuronal counting.

Fig. 2 illustrates the criteria used to distinguish ganglion cells, displaced amacrine cells, and other cells found in the platyrrhine ganglion cell layer. Fig. 2A shows the ganglion cell layer of a retinal flat mount from a *Cebus* monkey, 1.8 mm temporal to the fovea. Ganglion cells were labeled by biotinyl-lysine (Biocytin; Sigma, St. Louis, Missouri, USA) [40]. All retrograde labeled cells were ganglion cells and they were showed as black stained cell bodies (axons and dendrites were also labeled but are largely out of focus in this photomicrograph). Other cell types were never labeled and appeared as light brown cell bodies stained by the weak unspecific DAB reaction intensified by the osmium tetroxide exposure. They comprised displaced amacrine cells, displaced horizontal cells, microglia, astrocytes, and endothelial cells. Some ganglion cells were also not labeled once their axons were preserved by the incision to deposit the neurotracer. Careful inspection of a collection of retinal whole mounts from *Cercopithecus aethiops*, *Cebus apella*, and *Aotus trivirgatus* labeled by this procedure using Biocytin or horseradish peroxidase (HRP) was used to consolidate the criteria to identify and count ganglion cells and displaced amacrine cells in platyrrhines [29]–[31].

We also had available to us a retina from one *Macaca fascicularis*, generously provided by Professor Alan Cowey (Department of Experimental Psychology, University of Oxford) in which one optic tract had been sectioned 13 months previously. This retina had bee prepared as a wholemount and stained with cresyl violet [41].

Fig. 2B–D shows the ganglion cell layer of an *Alouatta* retinal flat mount stained with cresyl violet. The retinal ganglion cell layer is shown at three increasing eccentricities located temporal to the fovea. Neurons were readily distinguishable from glial cells and endothelial cells by their morphology. Ganglion cells comprised a heterogeneous population of neurons and were distinguishable from other neurons of the ganglion cell layer by the abundant amount of Nissl substance in their cytoplasm. The large- to medium-sized ganglion cells had a conspicuous amount of Nissl substance in the cytoplasm, an eccentric nucleus, and a clear nucleolus. These cells were readily labeled with Biocytin or HRP. The smaller ganglion cells had eccentrically placed nuclei, small rim of cytoplasm with small amount of Nissl substance, and were retrogradely labeled with Biocytin or HRP. All the neuronal classes that were positively identified as ganglion cells by Biocytin or HRP retrograde labeling were not found in the ganglion cell layer after optic tract section.

Other neurons of the retinal ganglion cell layer comprised displaced amacrine cells and displaced horizontal cells, the later relatively rare in comparison with the former [42]–[44]. Displaced amacrine cells had small cell bodies, dark staining nuclei, rarely visible nucleoli, and scant cytoplasm. Glial cells comprised microglia and astrocytes that were generally located internally (microglia) or externally (astrocytes and microglia) to the retinal ganglion cells. The endothelial cells could be distinguished by forming walls of blood vessels that cross the microscope field. The cells of the ganglion cell layer or close to it that survived several months after optic tract section were of the same classes never labeled with Biocytin or HRP. In despite the progressive increase of ganglion cell density and the decrease of ganglion cell size, the criteria to distinguish between the cell classes of the retinal ganglion cell layer could be applied throughout the retinal flat mount, including regions close to the fovea [34], [41]. In the foveal slope, 4–7 cell strata compose the ganglion cell layer and careful focus through its depth was necessary to perform precise neuronal counting.

### Ganglion cell density along the horizontal and vertical meridians of the *Alouatta* retina

Five retinas were counted over their entire extent. An additional retina was partially analyzed once counts were performed in the midperipheral and peripheral regions, for eccentricities higher than 2–3 mm of distance from fovea. Table 2 shows retinal area measurements performed before and after histology. The mean and standard deviation values were, respectively: 661±74 mm^2^ (N = 4) and 641±62 mm^2^ (N = 6), respectively. The mean areal shrinkage in the four retinas that were measured before and after histology was 4.8±4.3% (range: 1 to 11%). As shrinkage occurred only along retinal borders and cuts performed to flat the retinas, no corrections were applied for cell density estimates obtained by cell counting.

**Table 2:** Retinal area before (N = 4) and after histological procedure (N = 6), retinal shrinkage due to histological procedure (N = 4), and totals for ganglion cells and displaced amacrine cells (N = 5).

Retina	Retinal Area (mm^2^)	Retinal Area (mm^2^)	Shrinkage (%)	Ganglion Cells (Total)	Displaced Amacrine Cells (Total)
	Before	After			
AC 01LM	-	690	-	1,034,015	542,157
AC 02LM	-	644	-	1,180,200	639,500
AC 03LM	642	573	11	1,231,000	519,294
AC 04LM	770	736	4	1,146,683	478,685
AC 05LM	616	607	1	1,075,727	504,949
AC 05RM	615	595	3	-	-
Mean	661	641	4.8	1,133,525	536,917
Standard deviation	74	62	4.3	79,208	61,794
“Average” retina	-	644	-	1,078,968	519,970

Totals for ganglion cells and displaced amacrine cells were estimated by integration of isodensity contours. For the “average” retina, the isodensity contours were drawn from mean density values of N = 6 retinas and plotted in the map of retina AC 02LM. AC, *Alouatta caraya*. R, right retina. L, left retina. M, retina from a male howler monkey.

**Figure 3:**
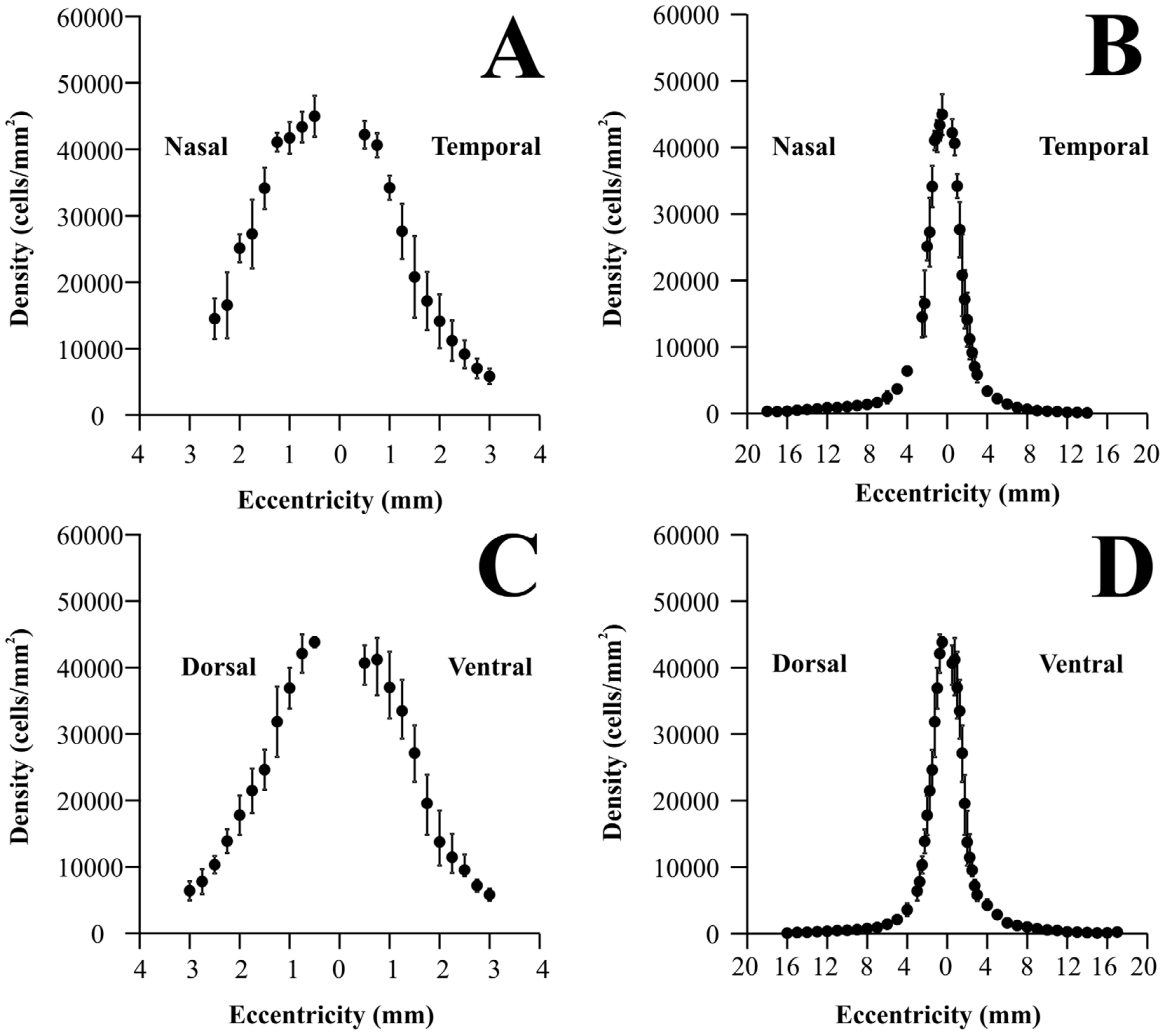
Ganglion cell density in the *Alouatta* retina. Ganglion cell density values were plotted along the nasotemporal horizontal (A–B) and dorsoventral vertical (C–D) meridians, respectively. Eccentricity values were taken as linear distances from the foveal pit. Left panels (**A** and **C**) show ganglion cell density for the central 3 mm of eccentricity, while right panels show values for the entire horizontal and vertical meridians (**B** and **D**). Filled circles and vertical bars represent means and standard deviations obtained from five retinas (central region) and six retinas (midperipheral and peripheral regions), respectively. Ganglion cell density reached maximum values in the foveal slope: 44963±3089, 42191±2063, 43825±788, and 40673±2669 cells/mm^2^ at 0.5 mm nasal, temporal, dorsal, and ventral to the fovea, respectively. Towards retinal periphery, ganglion cell density steeply declined reaching very low values: 274±131, 146±111, 166±63, and 97±78 cells/m^2^ in the nasal, temporal, dorsal, and ventral periphery, respectively. Ganglion cell density was higher in the nasal than in the temporal side at similar eccentricities, the nasal/temporal ration increased from 1.06 at 0.5 mm to 6.8 at 12 mm of eccentricity, respectively. There was no consistent difference in the dorsal versus ventral ganglion cell density.

Fig. 3 shows how ganglion cell density changed along the horizontal (A and B) and vertical (C and D) meridians of the *Alouatta* retina, respectively. Ganglion cell counts were converted to ganglion cells per square millimeter and eccentricity values were taken as distances from the foveal pit. Ganglion cell density means and standard deviations are shown in expanded plots for the central 3 mm of eccentricity (A and C) (N = 5) as well as for the entire range of eccentricities (B and D) (N = 6). Ganglion cell density reached maximum values in the foveal slope: 44,963±3,089, 42,191±2,063, 43,825±788, and 40,673±2,669 cells/mm^2^ at 0.5 mm nasal, temporal, dorsal, and ventral to the fovea, respectively. Towards the retinal periphery, ganglion cell density steeply declined to the very low values: 274±131, 146±111, 166±63, and 97±78 cells/m^2^ in the nasal, temporal, dorsal, and ventral periphery, respectively. Ganglion cell density was higher in the nasal than in the temporal side at similar eccentricities. The nasal/temporal ration increased from 1.06 at 0.5 mm to 6.8 at 12 mm of eccentricity. There was no consistent difference in the dorsal versus ventral ganglion cell density.

### Ganglion cell isodensity maps for the *Alouatta* retina

**Figure 4:**
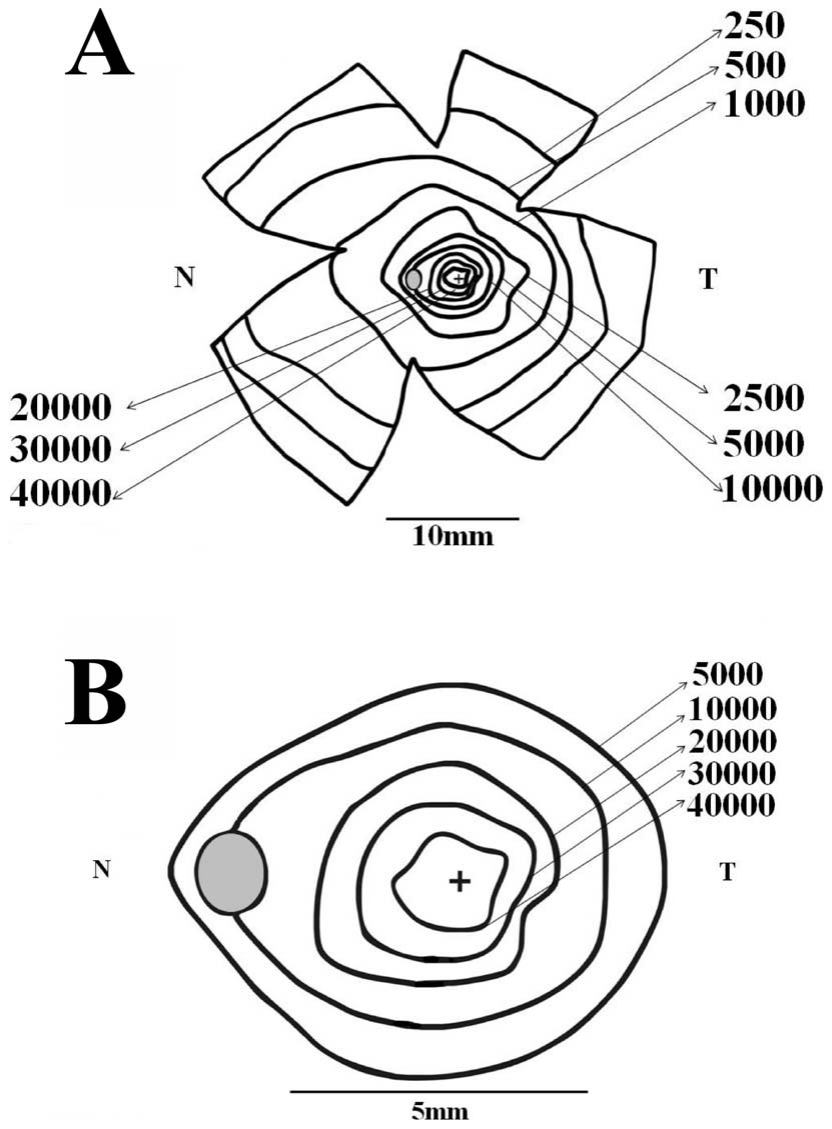
Ganglion cell isodensity maps for an *Alouatta* retina. (**A**) Isodensity contours for the retina AC 01 Left Male. (**B**) Isodensity contours for the central region of the same retina. Isodensity contours in cells/mm^2^ were drawn from cell density values estimated at every retinal location that was measured and displayed as if observing from the internal retinal side. A cross and a gray oval indicate the fovea and optic disk locations. N and T indicate the nasal and temporal directions along the horizontal meridian. In this retina and all the others that were analyzed, ganglion cell isodensity contours were slightly elongated in the nasal direction.

**Figure 5:**
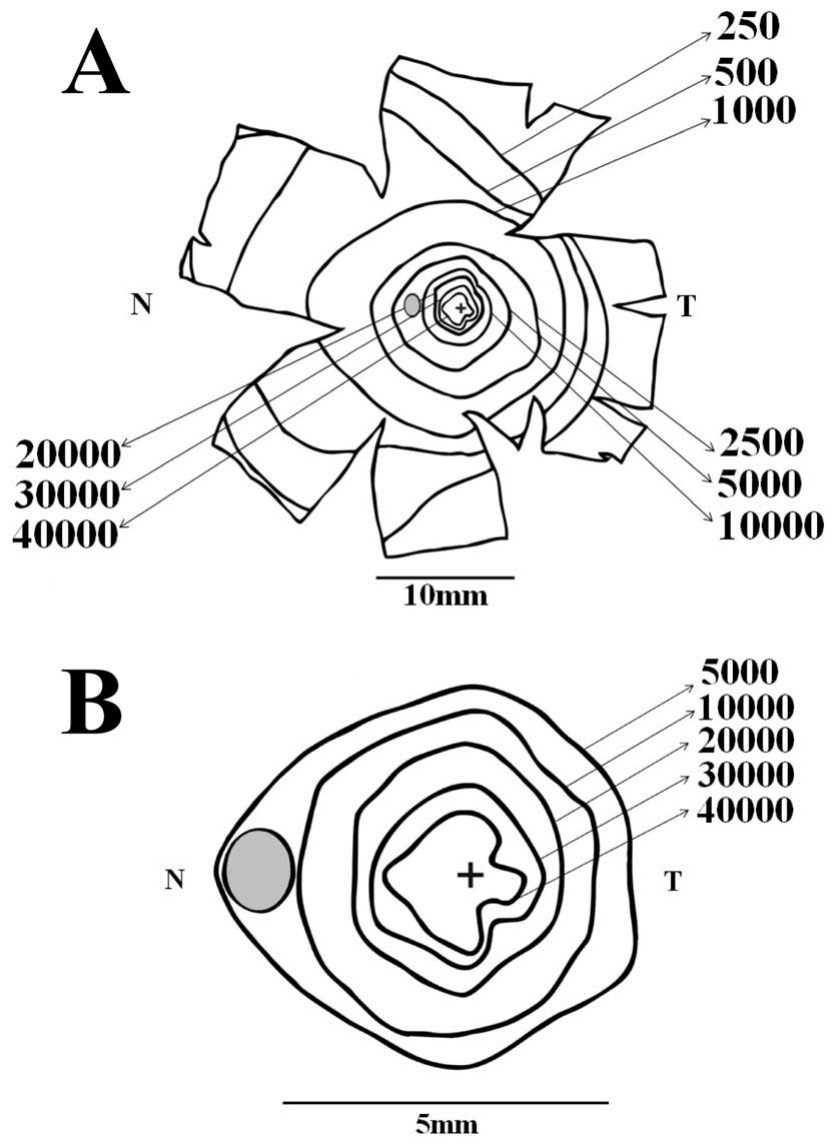
Ganglion cell isodensity maps for another *Alouatta* retina. (**A**) Isodensity contours for the retina AC 02 Left Male. (**B**) Isodensity contours for the central retinal region. Conventions were the same of Fig. 4. Similarly to other retinas studied in this work, ganglion cell isodensity contours were slightly elongated in the nasal direction. In addition, in this retina there was also an elongation of the central isodensity contours along the dorsoventral meridian.

Figs. 4–5 show ganglion cell isodensity maps for two *Alouatta* retinas. In every figure, the upper map shows isodensity contours for the whole retina, while the lower map shows isodensity contours for the central region at a larger magnification. Isodensity contours were drawn from cell density values estimated at every retinal location that was measured and were exhibited as if observing from the internal retinal side. Ganglion cell density was expressed in cells/mm^2^. A cross and a gray oval indicated the locations of the fovea and optic disk, respectively. N and T indicate the nasal and temporal directions along the horizontal meridian. In the first retina (AC 01LM), the ganglion cell isodensity contours were slightly elongated in the nasal direction (Fig. 4). In the second retina (AC 02LM), there was also an elongation of the central isodensity contours along the vertical meridian (Fig. 5).

**Figure 6:**
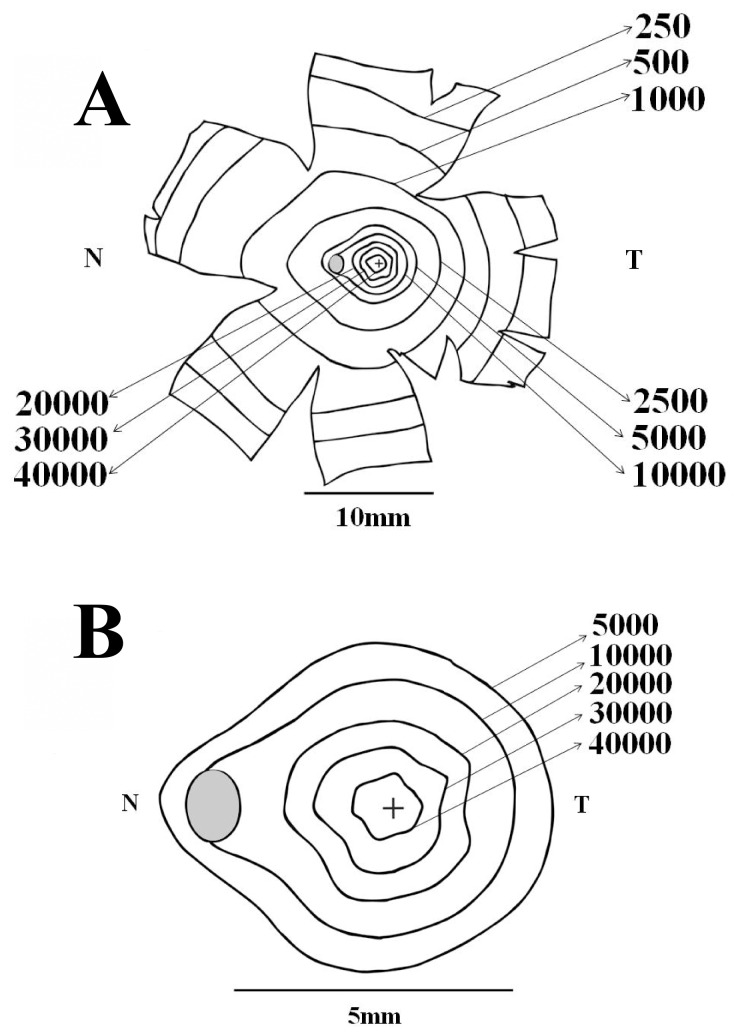
Ganglion cell isodensity maps for an “average” *Alouatta* retina. Isodensity contours in cells/mm^2^ were drawn from mean values from every retinal location that was measured and using the map of retina AC 02LM as template. (**A**) Isodensity contours for the whole retina. (**B**) Isodensity contours for the central retinal region. Conventions were the same of Figs. 4–5. Ganglion cell isodensity contours were elongated in the nasal direction reflecting the higher ganglion cell density in the nasal quadrant, a feature present in different degrees in all six retinas studied. The “average” retina also had a small elongation of the central isodensity contours along the dorsoventral meridian, reflecting the presence of this feature in half of the retinas (see text for details).

Fig. 6 shows an “average” ganglion cell isodensity map for the *Alouatta* retina. For these maps, the isodensity contours were drawn from cell density mean values estimated at every eccentricity that was studied and using the map of retina AC 02LM as template (N = 5 for the central region, N = 6 for midperipheral and peripheral regions). The ganglion cell isodensity contours were elongated in the nasal direction reflecting the higher ganglion cell density in the retinal nasal quadrant, a feature present in different degrees for all the six retinas. The “average” retina also had a small elongation of the central isodensity contours along the vertical meridian, reflecting the presence of this feature in half of the retinas.

### Displaced amacrine cell density along the horizontal and vertical meridians of the *Alouatta* retina

**Figure 7:**
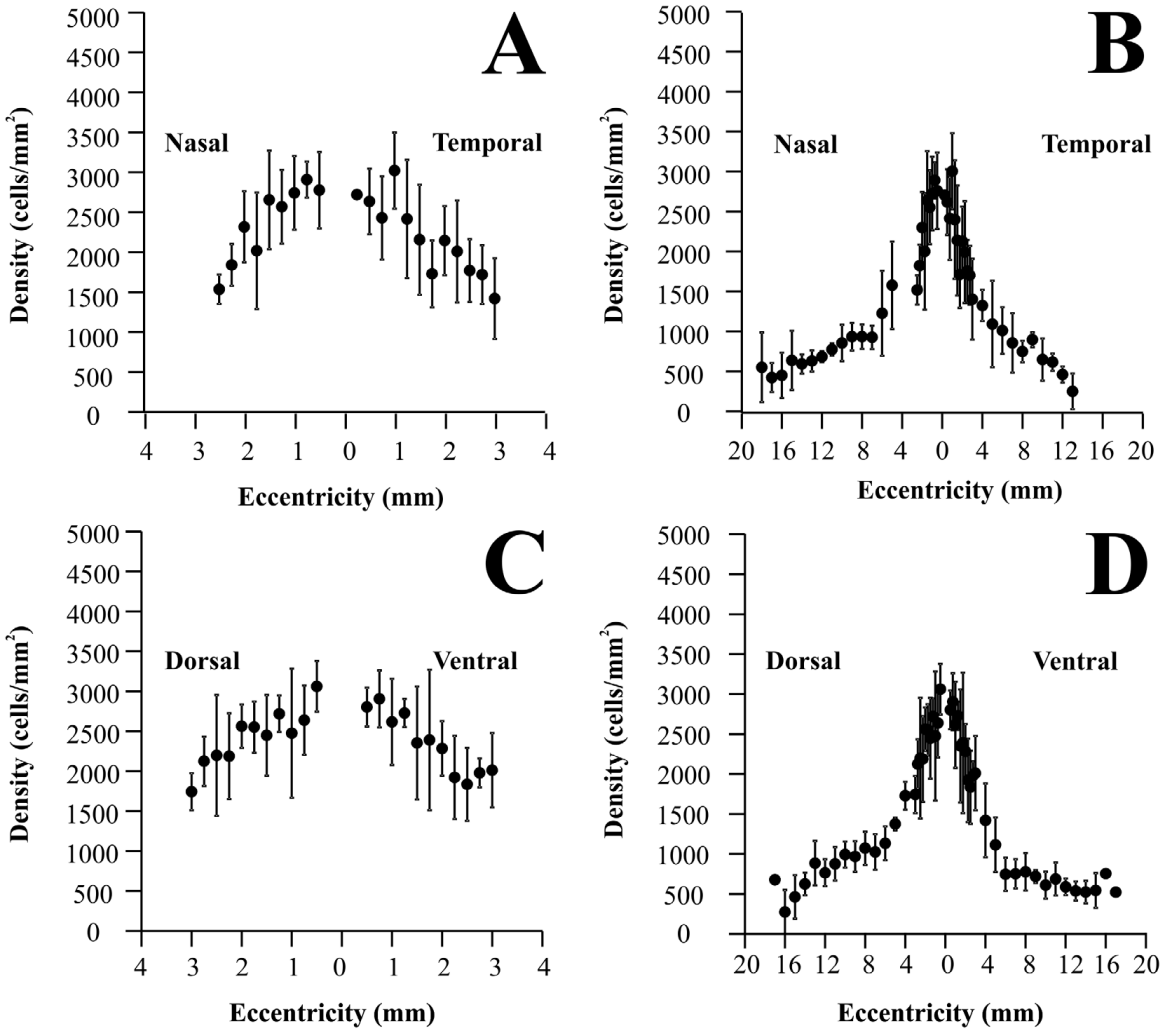
Displaced amacrine cell density in the *Alouatta* retina. Displaced amacrine cell density values were plotted along the nasotemporal horizontal (**A–B**) and dorsoventral vertical (**C–D**) meridians, respectively. Eccentricity values represent linear distances from the foveal pit. Left panels (**A** and **C**) show displaced amacrine cell density for the central 3 mm of eccentricity, while right panels show values for the entire horizontal and vertical meridians (**B** and **D**). Filled circles and vertical bars represent means and standard deviations obtained from five retinas (central region) and six retinas (midperipheral and peripheral regions), respectively. Displaced amacrine cell density reached maximum values in the foveal slope at 0.5–0.75 mm of eccentricity: 2907±227, 2719±411, 3062±317, and 2905±355 cells/mm^2^ nasal, temporal, dorsal, and ventral to the fovea, respectively. Towards retinal periphery, displaced amacrine cell density initially declined at a fast rate and more slowly further in the periphery. There was no consistent density difference in the nasal versus temporal or dorsal versus ventral quadrants.

Fig. 7 shows the density of displaced amacrine cells along the horizontal (A and B) and vertical (C and D) meridians of the *Alouatta* retina, represented as described for retinal ganglion cells. Displaced amacrine cell density reached maximum values in the foveal slope at 0.5–0.75 mm of eccentricity: 2,907±227, 2,719±411, 3,062±317, and 2,905±355 cells/mm^2^ nasal, temporal, dorsal, and ventral to the fovea, respectively. Toward the retinal periphery, displaced amacrine cell density initially declined at a fast rate and more slowly further in the periphery, reaching between 500–550 cells/mm^2^. There was no consistent density difference in the nasal versus temporal or dorsal versus ventral quadrants.

The total of ganglion cell layer neurons reached maximum values in the foveal slope at 0.5–0.75 mm of eccentricity, between 43,500–47,900 cells/mm^2^. At the retinal periphery this total remained between 600–850 cells/mm^2^. In the central region of the retina, the displaced amacrine cells represented between 6% and 6.7% of the total of neurons, while in the peripheral region they represented between 67% and 84% of ganglion cell layer neurons.

### Displaced amacrine cell isodensity maps for the Alouatta retina

**Figure 8:**
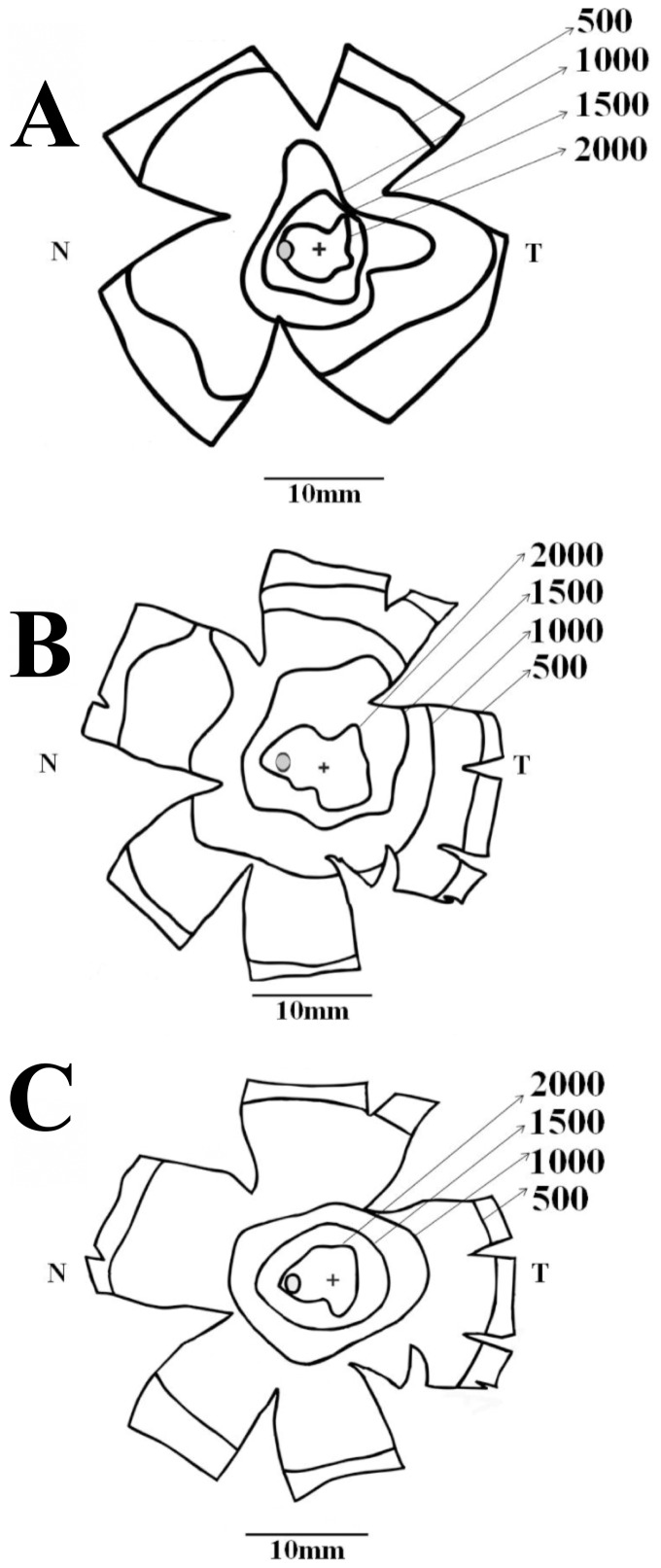
Displaced amacrine cell isodensity maps for the *Alouatta* retina. (**A**) Retina AC 01 Left Male. (**B**) Retina AC 02 Left Male. (**C**) “Average” retina using the map for AC 02LM as template. Conventions were the same of Figs. 4–5. Displaced amacrine cell density distribution followed a pattern approximately circular but with considerable variation in the central isodensity contours.

Fig. 8A–B shows displaced amacrine cell isodensity maps for two *Alouatta* retinas AC 01LM and AC 02LM, respectively. Fig. 8C shows the displaced amacrine cell isodensity map for the “average” *Alouatta* retina, using the AC 02LM retina as template. Maps were drawing as described above for the ganglion cells. Displaced amacrine cell density was expressed in cells/mm^2^. Displaced amacrine cell density distribution followed a pattern approximately circular but with considerable variation in the central isodensity contours.

### Total number of ganglion cells and displaced amacrine cells in the *Alouatta* retina

The number of ganglion cells and displaced amacrine cells were estimated by integration of the isodensity maps. Table 2 lists the results obtained for individual retinas and “average” retina. The total of ganglion cells ranged from 1,034,000 to 1,231,000 cells. The mean and standard deviation were 1,133,525±79,208 cells, respectively. The total of displaced amacrine cells ranged from 479,000 to 640,000 cells and the mean and standard deviation were 536,917±61,794 cells, respectively. The “average” retina had 1,598,938 ganglion cell layer neurons, corresponding to 1,078,968 ganglion cells and 519,970 displaced amacrine cells, respectively.

## Discussion

Measurement of the number and types of retinal neurons and photoreceptors, their distribution, the exact conformation of the eye, and the neuron number and volume of central visual system structures has long been used as a source of information about how visual systems may be specialized for particular niches, contrasted with what features are conserved across visual systems [45]–[50]. For primates, a growing corpus of information about the generation and maturation of the visual system has allowed insight into the nature of the mechanisms that generate basic retinal and central visual system organization, coordinate the number and distribution of photoreceptors, retinal neurons and central neurons in primates of varying eye and brain sizes, and differentiate the visual system into niche-specific variations [35]–[36], [51]–[54]. The singularity (thus far) of trichromacy in the *Alouatta* compared to other platyrrhines and its atypical fovea compared to all other diurnal Anthropoidea, could make the *Alouatta*'s visual system a naturally-occurring test of hypotheses about the role of the fovea in visual acuity and color vision. Though developmental data may be hard to acquire for this primate, the degree of disparity in density of cone packing within its fovea may be a natural test of several hypotheses of the mechanisms of foveal production [53], [55]–[56].

### Density distribution in platyrrhines and comparison with catarrhines

The topography of ganglion cell density and displaced amacrine cell density have been estimated in the retinas of several species of catarrhines and platyrrhines, either using retinal whole mounts alone or combining information gathered from retinal whole mounts and retinal sections. These studies have encompassed several genera of catarrhines and platyrrhines, including *Homo* [57]–[59], *Macaca* [41], [57], [60]–[62], *Cercopithecus* [63], *Papio* [64], *Cebus* [34], *Saimiri* [57], *Callithrix* [65], and *Aotus* [38], [66].

The ganglion cell density peak is similar in diurnal platyrrhines and catarrhines so far studied and is located in the foveal slope (Table 3). The values range from 30,000–63,000 ganglion cells/mm^2^, with the highest value observed in the retina of the *Callithrix jacchus jacchus* [65]. For the nocturnal platyrrhine *Aotus trivirgatus*, the peak of ganglion cell density is about one third lower than in diurnal platyrrhines and catarrhines (Table 3) and occurs in the center of an area centralis or in the slope of a rudimentary fovea [38], [66].

**Table 3:** Density peak of retinal ganglion cells in catarrhines and platyrrhines estimated by using different techniques. Data were rounded to the thousands. Estimates from retinal area are also listed.

Species	Retinal Area (mm^2^)	Retinal Ganglion Cells	Ref
		Density Peak	
		(cells/mm^2^)	
*Alouatta caraya*	641	43,000	*
*Cebus apella*	611	46,000	[34]
*Callithrix jacchus jacchus*	210	63,000	[65], [82]
*Aotus trivirgatus*	660	14,000	[38]
*Macaca mulatta*	670	33,000	41
*Macaca mulatta*	670	38,000	[41], [60]
*Macaca fascicularis*	527	48,000	[57], [62]
*Cercopithecus aethiops sabeus*	707	43,000	[63]
*Papio anubis*	793	30,000	[64]
*Homo sapiens*	1,012	35,000	[58]

*This work.

**Figure 9:**
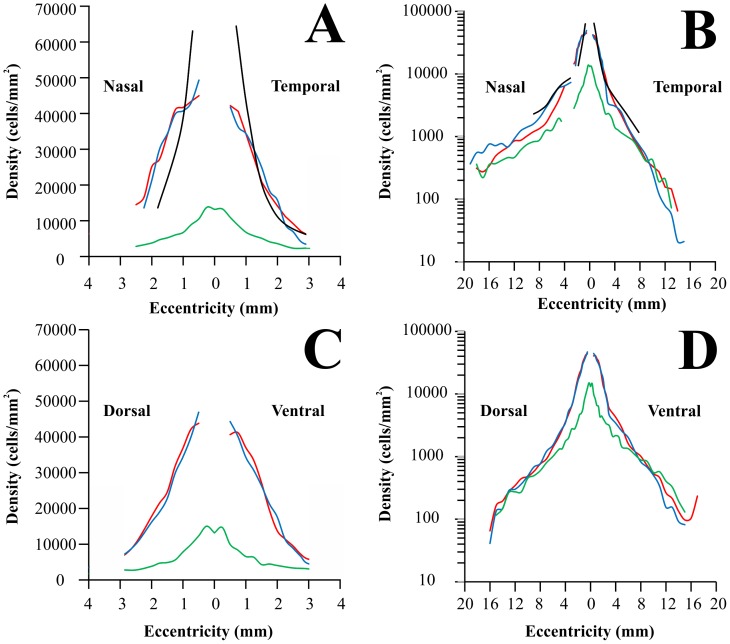
Retinal ganglion cell density in New World monkeys. Comparison of ganglion cell density in four different New World monkeys: *Alouatta caraya* (red lines; this study), *Cebus apella* (blues lines; Silveira et al., 1989a; only data from five male retinas from the original study were used in the plot), *Callithrix jacchus jacchus* (black lines; Wilder et al., 1996), and *Aotus trivirgatus* (green lines; Silveira et al., 1993). *Alouatta*, *Callithrix*, and *Cebus* are diurnals while *Aotus* is nocturnal. In addition, *Alouatta* (males and females) are trichromats, male *Cebus* and *Callithrix* are dichromats, and *Aotus* is monochromat. (**A–B**) Nasotemporal horizontal meridian. (**C–D**) Dorsoventral vertical meridian. Left panels (**A** and **C**) show ganglion cell density for the central 3 mm of eccentricity, whilst right panels show values for the entire horizontal and vertical meridians (**B** and **D**). Ganglion cell density in *Alouatta*, *Callithrix*, and *Cebus* are similar, but *Callithrix* has the highest peak density and the nasotemporal asymmetry is higher in *Cebus* and *Callithrix*, than *Alouatta*. In the central and intermediate retinal regions, ganglion cell density is higher in the diurnal monkeys, *Alouatta*, *Callithrix*, and *Cebus*, than in the nocturnal *Aotus*. In the region of highest ganglion cell density, there is a threefold difference, higher in the diurnal monkeys. In the retinal periphery, the four monkeys have approximately the same retinal ganglion cell density.

Fig. 9 shows how ganglion cell density changes in the retina of three platyrrhines of three different phenotypes, the diurnal trichromat *Alouatta caraya* (N = 5, this study; red lines), the diurnal dichromat *Cebus apella* (N = 5 male dichromats, blue lines) [34], and the nocturnal monochromat *Aotus trivirgatus* (N = 4, green lines) [38]. Data were collected using similar procedures and are presented along the horizontal (A and B) and vertical (C and D) meridians with two different resolutions in the horizontal axes. In the same graphs, we have also plotted data of ganglion cell density obtained by Wilder and colleagues, using different procedures, for another diurnal dichromatic platyrrhine, the common marmoset *Callithrix jacchus jacchus* [65]. *Callithrix* has a retina substantially smaller than *Alouatta*, *Cebus*, and *Aotus*, measuring about 200 mm^2^ versus 600–700 mm^2^ in the other three platyrrhines. Ganglion cell density in *Alouatta* and *Cebus* are largely similar. The ganglion cell density in *Callithrix* is higher than in *Cebus* and *Alouatta* in the foveal slope, becomes lower for intermediate eccentricities, and similar to the other two diurnal platyrrhines for more peripheral eccentricities.

The nasotemporal asymmetry is larger in *Cebus* and *Callithrix* than in *Alouatta*. The nasotemporal asymmetry is due to a horizontal specialization, the visual streak, superposed to a radial specialization, the fovea and area centralis. Visual streaks are found in the retinas of many mammals and are particularly well developed in lateral eyed mammals such as rabbit [67], agouti [48], and giraffe [68], among many others. They have been hypothesized to serve the vision of objects standing on the ground and located around in the horizon [45]–[46]. Some diurnal primates such as baboons (*Papio Anubis*) [64] and humans [57]–[58] have more pronounced visual streaks when compared with other primates, and this finding has been related to their ground dwelling/foraging and secondary terrestrial behavior [57]. Other diurnal catarrhines and platyrrhines have less pronounced visual streaks than baboons and humans, and this group comprises macaques (*Macaca mulatta* [41], *Cercopithecus aethiops sabeus* [63]), capuchin monkeys (*Cebus apella* [34]), squirrel monkeys (*Saimiri sciureus* [57], and marmosets (*Callithrix jacchus jacchus* [65]. The less distinctive nasotemporal asymmetry of howler monkeys compared with that exhibited by other diurnal species of higher primates may be related to their highly arboreal behavior. *Alouatta* are large and slow-moving monkeys eating mainly top canopy leaves, and at lesser extent fruits, buds, flowers, and nuts, carefully not eating too many leaves of certain species without stopping, as some contain poison toxins [69]. Most *Alouatta* species do prefer the main canopy and emergent levels, but some species, such as *Alouatta caraya*, living in less favorable environments, come to the ground and cross open areas between forest patches [69]. In closely related mammals, the visual streak is more conspicuous in those with diurnal behavior than in nocturnal ones [48]. Platyrrhines conform to this pattern, and the nasotemporal asymmetry is more pronounced in the diurnals *Cebus*, *Callithrix*, and *Alouatta* than in the nocturnal *Aotus*.

In conclusion, *Alouatta* follows the common pattern of retinal ganglion cell distribution found in all diurnal platyrrhines and catarrhines: a distinct fovea whose size is approximately constant, about 250–500 µm in diameter in different species [33]; a very high ganglion cell density in the foveal slope; a rudimentary visual streak, less marked than in some other diurnal platyrrhines and catarrhines; and a very low ganglion cell density in the retinal periphery, especially in the temporal, dorsal, and ventral quadrants. The only marked distinction *Alouatta* shows is a very high cone density in the central fovea, to be discussed.

### Retinal ganglion cell sampling density in platyrrhines and catarrhines

The complexity of the primate fovea, with the displacement of cone cell bodies and all other neurons, makes it difficult to measure how the ganglion cell sampling density changes as a function of eccentricity in perifoveal and foveal regions. Taking into account the changing densities of cones, cone pedicles, and ganglion cells in combination with the offsets introduced by lateral displacement of cone pedicles, bipolar cells, and ganglion cells in the foveal region, these calculations have been performed for *Macaca mulatta* [60], *Macaca fascicularis* [61]–[62], [70], *Callithrix jacchus jacchus* [65], and humans [58], [71]–[74]. The values for ganglion cell sampling density obtained in these studies range from 200,000 to 668,000 ganglion cells/mm^2^ for the ganglion cells connected to the most central cones, producing ratio of 1 cone to 2 or more ganglion cells thought to be critical in the evolution of trichromacy.

**Figure 10:**
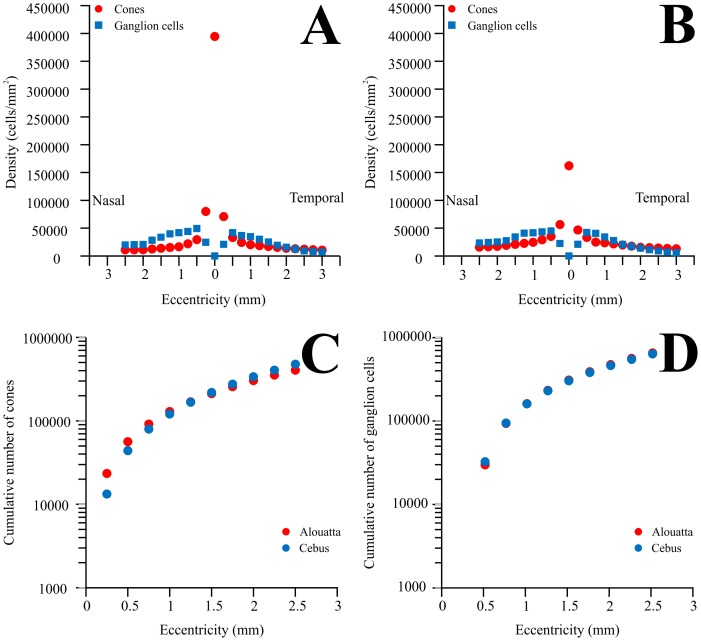
Comparison between the distributions of ganglion cells and cones in the central region of *Alouatta* and *Cebus* retinas. Trichromatic *Alouatta* differs from dichromatic male *Cebus* essentially by having high cone density in the foveola. The plots show that this occurs at expenses of a decrease in the number of cones in the remaining of the central region of the *Alouatta* retina. Other aspects of the retinal distribution of cones and ganglion cells are essentially similar in the trichromatic *Alouatta* and dichromatic *Cebus*. (**A**) Density of cones [33] and ganglion cells (this study) across the horizontal meridian of the *Alouatta* retina. (**B**) Density of cones [33] and ganglion cells [34] across the horizontal meridian of the *Cebus* retina. Only data from male dichromats were plotted. (**C**) Cumulative number of cones in the retinas of *Alouatta* and *Cebus*. Data from the original study [33] were used to estimate the total number of cones in ring of retina centered in the foveola. Then, the cumulative number of cones in circular areas was estimated by adding the number of cones in successive rings. (**D**) Cumulative number of ganglion cells in the retinas of *Alouatta* and *Cebus* obtained from ganglion cell densities estimated in this study and the original study of Silveira and colleagues [34]. The cumulative number of ganglion cells in circular retinal areas was estimated by adding the number of ganglion cells in successive rings centered in the foveola.

While the detailed anatomy used in the prior studies is beyond the scope of the present study, overall calculations can be made to determine if the high peak cone density in *Alouatta* results in higher total cone numbers in its fovea compared to *Cebus* (Fig. 10). Considering variable extents of the fovea, taking cone numbers from prior studies [33], [35], integrating over a region 1 mm diameter in the foveal center would contain approximately 44,000 cones in *Cebus* and 56,000 cones in *Alouatta* while integrating over a region of 4 mm diameter would reverse the relative magnitudes, producing about 338,000 cones in *Cebus* and 304,000 cones in *Alouatta*. Therefore, it seems to be the case that the entire foveal region does not contain more cones in *Alouatta*, but rather has a steeper gradient in cone density from the foveal central to its border, compared both to *Cebus* and other diurnal platyrrhines and catarrhines (Fig. 10). The similar foveal cone numbers will converge on ganglion cell densities and corresponding numbers that are similar (Fig. 10).

At this point, we can only speculate whether the high cone density in the central fovea of *Alouatta* can be employed to increase the spatial sampling of the retinal ganglion cells representing the very central fovea, and whether this feature plays some role in the evolution of its trichromacy. The tight packing and reduced diameter of the central cone outer segments might suggest that the fovea had been produced over a somewhat longer time in *Alouatta* (perhaps beginning earlier), which would allow more time for the compaction of the central cones. Alternatively, perhaps some feature of the altered opsins themselves in this radiation permits greater variation in cone morphology.

### Displaced amacrine cell density distribution in platyrrhines

**Figure 11:**
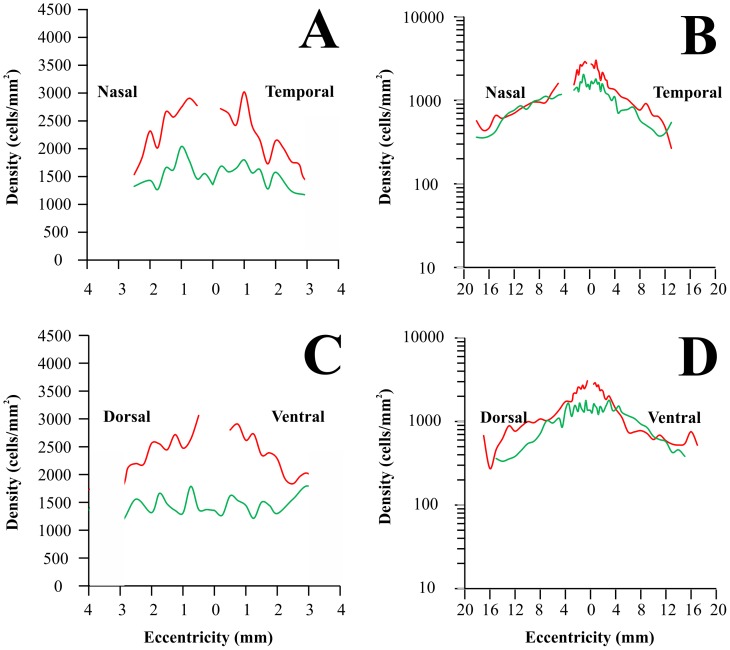
Displaced amacrine cell density in New World monkeys. Comparison of displaced amacrine cell density in two different New World monkeys: *Alouatta* (red lines, this study) and *Aotus* (green lines, [38]). *Alouatta* is diurnal and trichromat while *Aotus* is nocturnal and monochromat. (**A–B**) Nasotemporal horizontal meridian. (**C–D**) Dorsoventral vertical meridian. Displaced amacrine cell counts were converted to cells per square millimeter and eccentricity values were taken as distances from the foveal pit. Left panels (**A** and **C**) show displaced amacrine cell density for the central 3 mm of eccentricity, whilst right panels show values for the entire horizontal and vertical meridians (**B** and **D**). The displaced amacrine cell density is higher in the diurnal *Alouatta* than in the nocturnal *Aotus* in the central retinal region and most of retinal periphery.

Fig. 11 compares how displaced amacrine cell density changes in the retina of the *Alouatta* (red lines, this study) and *Aotus* (green lines [38]) along the horizontal (A and B) and vertical (C and D) meridians. Plots are presented in two magnifications. The displaced amacrine cell density is higher in the diurnal trichromat *Alouatta* than in the nocturnal monochromat *Aotus* in the central retinal region and most of retinal periphery. However, the difference between the two platyrrhines is smaller than that observed for retinal ganglion cells in the central region, being less than two times for displaced amacrine cells and about three times for ganglion cells. The difference of the centroperipheral gradient is also smaller for displaced amacrine cells than for ganglion cells in these two platyrrhines.

The totals of displaced amacrine cells estimated in five *Alouatta* retinas in this study were listed in Table 2. The mean value found for the “average” retina was 520,000 displaced amacrine cells. Data about the totals of displaced amacrine cells in different primates are scarce. Silveira and colleagues reported individual values for single *Aotus* retinas as well as a total 444,000 displaced amacrine cells for the “average” *Aotus* retina (n = 4) [38]. Thus, is spite of having 2.4 times more ganglion cells than the nocturnal *Aotus*, the diurnal *Alouatta* has only 1.2 times more displaced amacrine cells.

The *Aotus* has developed a fully nocturnal retina in recent evolution, while *Alouatta* has all the features of a well-developed diurnal retina ([35], [53], this work). Finlay and colleagues have proposed the hypothesis that conserved developmental programs, such as the order of neurogenesis in the mammalian eye, might explain the relative numbers of different neuronal populations in the primate retina and suggested that they are useful features for evolutionary stability and variability [35], [53], [75]. Description and quantification of cell cycle kinetics show that embryonic cytogenesis is extended in *Aotus* compared with the diurnal New World monkey *Cebus* [36]. Combined with the conserved mammalian pattern of retinal cell specification [76]–[78], this single change in retinal progenitor cell proliferation can produce the multiple alterations of the nocturnal *Aotus* retina, including coordinated reduction in the numbers of early generated neurons such as cone and ganglion cells, moderate increase in the numbers of horizontal cells and amacrine cells, and increase in the numbers of late generated neurons such as rod and rod bipolar cells [36].

### Total number of ganglion cells in the *Alouatta* retina, and in primates generally

**Figure 12:**
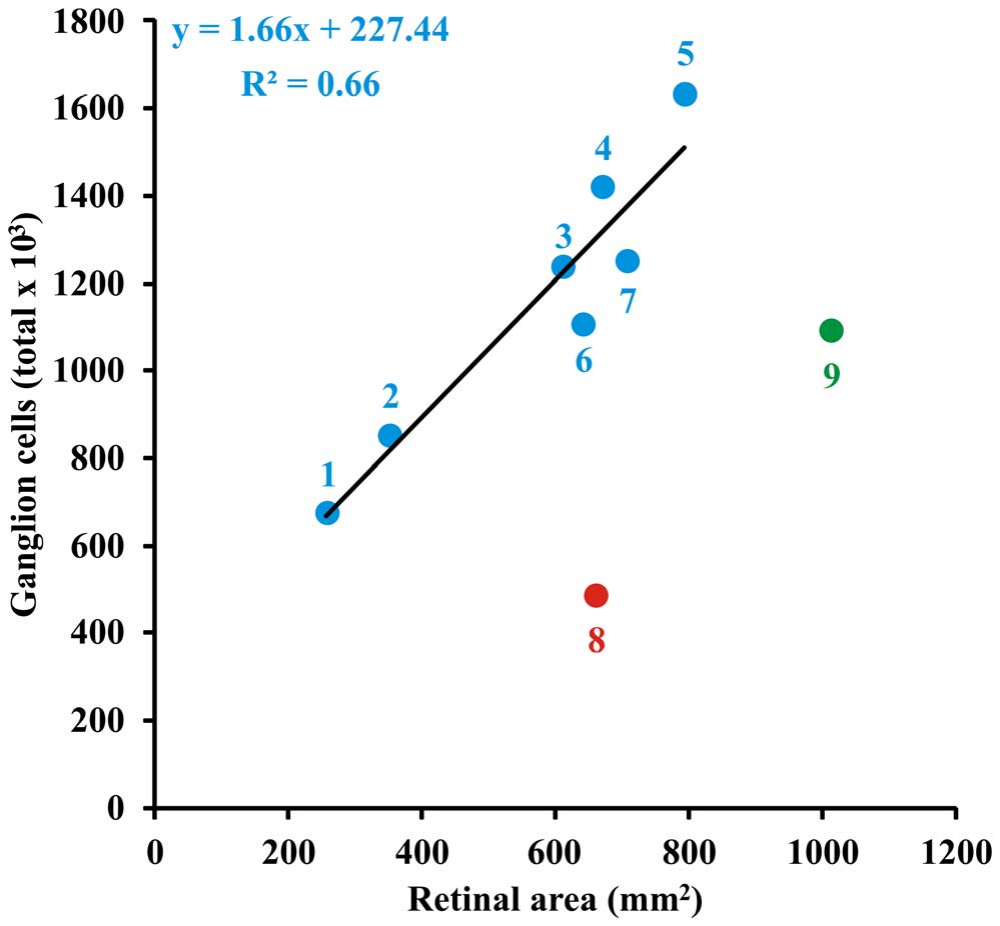
Relation between total of ganglion cells and retinal area in different species of primates. Data were grand means across different studies (Table 3). The following catarrhines and platyrrhines were represented in the plot: *Saguinus midas niger* (1), *Saimiri sciureus* (2), *Cebus apella* (3), *Macaca mulatta* (4), *Papio anubis* (5), *Alouatta caraya* (6), *Cercopithecus aethiops sabeus* (7), *Aotus trivirgatus* (8), and *Homo sapiens* (9). Only data from diurnal species of monkeys were considered in the linear regression fitting (blue circles). Data from the single nocturnal species (*Aotus*, red circle) as well as data from humans (green circle) were excluded from the analysis. There was a good correlation for diurnal monkeys between the total of ganglion cells estimated by counting retinal ganglion cell bodies or optic nerve axons and retinal area. *Alouatta* had the number of ganglion cells that it was expected by its retinal area (see the text for further comments).

The two methods of calculating ganglion cell numbers, from counts of cell bodies in whole mounts, and from estimation of optic nerve axon numbers converge closely: 1,133,525±79,208 ganglion cell bodies (Table 2) and 1,081,000±91,989 optic nerve axons, respectively, giving our final estimation of 1,110,181±84,122 ganglion cells. Estimates for the total of ganglion cells are also available for other platyrrhines and also for catarrhines, obtained by a variety of procedures applied to either retinal ganglion cell bodies or optic nerve axons, and are summarized in Table 4. Fig. 12 illustrates the relation between total of ganglion cells and retinal area in platyrrhines and catarrhines. The values plotted were grand means across the studies listed in Table 4. Only data from diurnal species of monkeys were considered in the linear regression fitting. This extends and confirms prior observations on the close relationships of retinal area, photoreceptor and retinal neuron number, and brain volume in diurnal primates, with the notable exception of humans [35]. The nocturnal *Aotus* also has reduced numbers of ganglion cells, which we have discussed in detail elsewhere [35]–[36].

**Table 4:** Total of retinal ganglion cell bodies or optic nerve axons in catarrhines and platyrrhines estimated by using different techniques. Data were rounded to the thousands. Estimates from retinal area are also listed.

Species	Retinal Area (mm^2^)	Retinal Ganglion Cells (Total)	N	Optic Nerve Axons (Total)	N	Ref
*Alouatta caraya*	641	1,134,000	5	-	-	*
*Alouatta caraya*	-	-	-	1,081,000	4	[35]
*Cebus apella*	611	1,400,000	6	-	-	[34]
*Cebus apella*	565	-	-	1,077,000	1	[35]
*Saimiri ustius*	352	-	-	853,000	4	[35]
*Callicebus moloch*	-	-	-	701,000	2	[35]
*Saguinus midas niger*	258	-	-	677,000	2	[35]
*Aotus trivirgatus*	660	479,000	4	-	-	[38]
*Aotus azarae*	595	-	-	496,000	3	[35]
*Macaca mulatta*	-	-	-	1,230,000	2	[83]
*Macaca mulatta*	-	-	-	1,410,000	1	[84]
*Macaca mulatta*	-	-	-	1,621,000	3	[85]
*Macaca mulatta*	-	-	-	1,250,000	2	[86]
*Macaca mulatta*	670	1,600,000	2	-	-	[41]
*Cercopithecus aethiops sabeus*	707	1,227,000	5	1,276,000	5	[63]
*Papio Anubis*	793	1,478,000	4	1,789,000	2	[64]
*Homo sapiens*	-	-	-	1,270,000	6	[87]
*Homo sapiens*	-	-	-	1,010,000	10	[83]
*Homo sapiens*	-	1,222,000	5	-	-	[88]
*Homo sapiens*	-	-	-	1,100,000	1	[89]
*Homo sapiens*	-	-	-	1,218,000	2	[85]
*Homo sapiens*	-	-	-	964,000	5	[90]
*Homo sapiens*	-	-	-	1,244,000	16	[91]
*Homo sapiens*	-	-	-	1,084,000	13	[92]
*Homo sapiens*	-	-	-	693,000	19	[93]
*Homo sapiens*	1,012	1,070,000	4	-	-	[58]
*Homo sapiens*	-	-	-	1,158,000	72	[94]

*This work.

Given the general belief in the primacy and superiority of vision in humans, and the general predictability of allometric scaling, this deviation in humans comes as a surprise, and we take this opportunity to propose a hypothesis to account for it. Perhaps the combination of a large brain and slightly lower ganglion cell numbers in humans suggests that computation can substitute for evolutionarily-difficult improvements in optical quality and cell-density-based increased acuity. Adding more resolution at the high frequency end of the spectrum becomes progressively more expensive per information gained in terms of retinal processing resources, because of the increasingly unfavorable noise-to-signal ratio at high frequencies. Contour integration and other aspects of environmental prediction may normally depend on integration over multiple sources of information at multiple neural levels [79], and optimal coding can employ the co-variation between these multiple sources to perform better its predictions of the source signal even if sampling frequency is low [80]. For an empirical example, in normal aging, increasingly better environmental prediction gained over long periods has been shown to reduce the requirements for immediate sensory learning [81]. Changing computational resources as well as the design of sensory end-organs may need to be incorporated into our understanding of the evolution of sensory systems.

## Supporting Information

S1 Appendix Supporting *Alouatta* housing conditions including feeding regimens and environmental enrichment.
(DOC)

S1 Checklist The ARRIVE Guidelines Checklist.
(DOC)

S1 Dataset Ganglion cell density, displaced amacrine cell density, total number of ganglion cells, cumulative number of cones and ganglion cells.
(ZIP)

## References

[1] Nathans J, Thomas D, Hogness DS (1986) Molecular genetics of human colour vision: the genes encoding blue, green and red pigments. Science 232:193–202. 2937147 10.1126/science.2937147

[2] Bowmaker JK, Astell S, Hunt DM, Mollon JD (1991) Photosensitive and photostable pigments in the retinae of Old World monkeys. J Exp Biol 156:1–19. 2051127 10.1242/jeb.156.1.1

[3] Ibbotson RE, Hunt DM, Bowmaker JK, Mollon JD (1992) Sequence divergence and copy number of the middle– and long–wave photopigment genes in Old World monkeys. Proc R Soc Lond B 247:145–154. 10.1098/rspb.1992.0021 1349182

[4] Polyak SL (1941) The Retina. Chicago, Illinois, USA: The University of Chicago Press, 721 p.

[5] Boycott BB, Wässle H (1991) Morphological classification of bipolar cells of the primate retina. Eur J Neurosci 3:1069–1088. 12106238 10.1111/j.1460-9568.1991.tb00043.x

[6] de Monasterio FM, Gouras P (1975) Functional properties of ganglion cells of the rhesus monkey retina. J Physiol (Lond) 251:167–195. 810576 10.1113/jphysiol.1975.sp011086 PMC1348381

[7] de Monasterio FM, Gouras P, Tolhurst DJ (1975a) Trichromatic colour opponency in ganglion cells of the rhesus monkey retina. J Physiol (Lond) 251:197–216. 810577 10.1113/jphysiol.1975.sp011087 PMC1348382

[8] de Monasterio FM, Gouras P, Tolhurst DJ (1975b) Concealed colour opponency in ganglion cells of the rhesus monkey retina. J Physiol (Lond) 251:217–229. 810578 10.1113/jphysiol.1975.sp011088 PMC1348383

[9] Lee BB, Martin PR, Valberg A (1988) The physiological basis of heterochromatic flicker photometry demonstrated in the ganglion cells of the macaque retina. J Physiol (Lond) 404:323–347. 3253435 10.1113/jphysiol.1988.sp017292 PMC1190828

[10] Lee BB, Martin PR, Valberg A (1989a) Sensitivity of macaque retinal ganglion cells to chromatic and luminance flicker. J Physiol (Lond) 414:223–243. 2607430 10.1113/jphysiol.1989.sp017685 PMC1189139

[11] Lee BB, Martin PR, Valberg A (1989b) Amplitude and phase of responses of macaque retinal ganglion cells to flickering stimuli. J Physiol (Lond) 414:245–263. 2607431 10.1113/jphysiol.1989.sp017686 PMC1189140

[12] Hunt DM, Dulai KS, Cowing JA, Julliot C, Mollon JD, et al (1998) Molecular evolution of trichromacy in primates. Vision Res 38:3299–3306. 9893841 10.1016/s0042-6989(97)00443-4

[13] Jacobs GH (2007) New World monkeys and color. Int J Primatol 28:729–759.

[14] Wikler KC, Rakic P (1990) Distribution of photoreceptor subtypes in the retina of diurnal and nocturnal primates. J Neurosci 10:3390–3401. 2145402 10.1523/JNEUROSCI.10-10-03390.1990 PMC6570186

[15] Jacobs GH, Neitz M, Neitz J (1996a) Mutations in S-cone pigment genes and the absence of colour vision in two species of nocturnal primate. Proc R Soc Lond B 263:705–710. 10.1098/rspb.1996.0105 8763792

[16] Silveira LCL, Lee BB, Yamada ES, Kremers J, Hunt DM (1998) Post-receptoral mechanisms of colour vision in New World primates. Vision Res 38:3329–3337. 9893845 10.1016/s0042-6989(97)00335-0

[17] Chan TL, Martin PR, Clunas N, Grünert U (2001) Bipolar cell diversity in the primate retina: morphologic and immunocytochemical analysis of a New World monkey, the marmoset *Callithrix jacchus* . J Comp Neurol 437:219–239. 11494253 10.1002/cne.1280

[18] Lee BB, Silveira LCL, Yamada ES, Kremers J (1996) Parallel pathways in the retina of Old and New World primates. Rev Brasil Biol 56 (Supl 1):323–338. 9394511

[19] Lee BB, Silveira LCL, Yamada ES, Hunt DM, Kremers J, et al (2000) Visual responses of ganglion cells of a New World primate, the capuchin monkey, *Cebus apella* . J Physiol (Lond) 528:573–590. 11432364 10.1111/j.1469-7793.2000.00573.x PMC2270155

[20] Silveira LCL, Lee BB, Yamada ES, Kremers J, Hunt DM, et al (1999) Ganglion cells of a short wavelength sensitive cone pathway in New World monkeys: morphology and physiology. Vis Neurosci 16:333–343. 10367967 10.1017/s0952523899162138

[21] Jacobs GH, Neitz M, Deegan JF, Neitz J (1996b) Trichromatic colour vision in New World monkeys. Nature 382:156–158. 8700203 10.1038/382156a0

[22] Araújo AC, Didonet JJ, Araújo CS, Saletti PG, Borges TR, et al (2008) Color vision in the black howler monkey (*Alouatta caraya*). Vis Neurosci 25:243–248. 18598395 10.1017/S0952523808080292

[23] dos Reis JWL, de Carvalho WA, Saito CA, Silveira LCL (2002) The morphology of horizontal cells in the retina of the capuchin monkey, *Cebus apella*: how many horizontal cell classes are found in dichromatic primates? J Comp Neurol 443:105–123. 11793350

[24] Silveira LCL, Yamada ES, Perry VH, Picanço-Diniz CW (1994) M and P retinal ganglion cells of diurnal and nocturnal New-World monkeys. NeuroReport 5:2077–2081. 7865749 10.1097/00001756-199410270-00022

[25] Ghosh KK, Goodchild AK, Sefton AE, Martin PR (1996) The morphology of retinal ganglion cells in the New World marmoset monkey *Callithrix jacchus* . J Comp Neurol 366:76–92. 8866847 10.1002/(SICI)1096-9861(19960226)366:1<76::AID-CNE6>3.0.CO;2-H

[26] Ghosh KK, Martin PR, Grünert U (1997) Morphological analysis of the blue cone pathway in the retina of a New World monkey, the marmoset *Callithrix jacchus* . J Comp Neurol 379:211–225. 9050786

[27] Ghosh KK, Grünert U (1999) Synaptic input to small bistratified (blue-on) ganglion cells in the retina of a New World monkey, the marmoset *Callithrix jacchus* . J Comp Neurol 413:417–428. 10502249 10.1002/(sici)1096-9861(19991025)413:3<417::aid-cne5>3.0.co;2-h

[28] de Lima SMA, Silveira LCL, Perry VH (1996) Distribution of M retinal ganglion cells in diurnal and nocturnal New-World monkeys. J Comp Neurol 368:538–552. 8744442 10.1002/(SICI)1096-9861(19960513)368:4<538::AID-CNE6>3.0.CO;2-5

[29] Yamada ES, Silveira LCL, Perry VH (1996a) Morphology, dendritic field size, somal size, density and coverage of M and P retinal ganglion cells of dichromatic *Cebus* monkeys. Vis Neurosci 13:1011–1029. 8961532 10.1017/s0952523800007677

[30] Yamada ES, Silveira LCL, Gomes FL, Lee BB (1996b) The retinal ganglion cell classes of New World primates. Rev Brasil Biol 56 Suppl 1: 381–396. 9394516

[31] Yamada ES, Silveira LCL, Perry VH, Franco ECS (2001) Morphology and dendritic field size of M and P retinal ganglion cells of the owl monkey. Vision Res 41:119–131. 11163848 10.1016/s0042-6989(00)00244-3

[32] Gomes FL, Silveira LCL, Saito CA, Yamada ES (2005) Density, proportion, and dendritic coverage of retinal ganglion cells of the common marmoset (*Callithrix jacchus jacchus*). Brazil J Med Biol Res 38:915–924. 10.1590/s0100-879x2005000600014 15933786

[33] Franco ECS, Finlay BL, Silveira LCL, Yamada ES, Crowley JC (2000) Conservation of absolute foveal area in New World primates: a constraint on eye size and conformation. Brain Behav Evol 56:276–286. 11251320 10.1159/000047211

[34] Silveira LCL, Picanço-Diniz CW, Sampaio LFS, Oswaldo-Cruz E (1989a) Retinal ganglion cell distribution in the *Cebus* monkey: a comparison with the cortical magnification factors. Vision Res 29:1471–1483. 2635473 10.1016/0042-6989(89)90131-4

[35] Finlay BL, Franco ECS, Yamada ES, Crowley JC, Parsons MP, et al (2008) Number and topography of cones, rods and optic nerve axons in New and Old World primates. Vis Neurosci 25:289–299. 18598400 10.1017/S0952523808080371

[36] Dyer MA, Martins R, da Silva Filho M, Muniz JAPC, Silveira LCL, et al (2009) Developmental sources of conservation and variation in the evolution of the primate eye. Proc Natl Acad Sci USA 106:8963–8968. 19451636 10.1073/pnas.0901484106 PMC2690025

[37] Curcio CA, Sloan KR, Kalina RE, Hendrickson AE (1990) Human photoreceptor topography. J Comp Neurol 292:497–523. 2324310 10.1002/cne.902920402

[38] Silveira LCL, Perry VH, Yamada ES (1993) The retinal ganglion cell distribution and the representation of the visual field in area 17 of the owl-monkey *Aotus trivirgatus* . Vis Neurosci 10:887–897. 8217938 10.1017/s095252380000609x

[39] Ayres M, Ayres Jr**.** M, Ayres DL, dos Santos AAS (2007) BioEstat 5.0: Aplicações Estatísticas nas Áreas das Ciências Biológicas e Médicas. Belém, Pará, Brazil: Instituto de Desenvolvimento Sustentável Mamirauá (IDSM/MCT/CNPq), 384 p.

[40] Picanço-Diniz CW, Silveira LCL, Yamada ES, Martin KAC (1992) Biocytin as retrograde tracer in mammal visual system. Brazil J Med Biol Res 25:57–62. 1304945

[41] Perry VH, Cowey A (1985) The ganglion cell and cone distributions in the monkey's retina: implications for central magnification factors. Vision Res 25:1795–1810. 3832605 10.1016/0042-6989(85)90004-5

[42] Silveira LCL, Yamada ES, Picanço-Diniz CW (1989b) Displaced horizontal cells and biplexiform horizontal cells in the mammalian retina. Vis Neurosci 3:483–488. 2487119 10.1017/s0952523800005988

[43] Wässle H, Dacey DM, Haun T, Haverkamp S, Grünert U, et al (2000) The mosaic of horizontal cells in the macaque monkey retina: With a comment on biplexiform ganglion cells. Vis Neurosci 17:591–608. 11016578 10.1017/s0952523800174097

[44] de Lima SMA, Ahnelt PK, Carvalho TO, Silveira JS, Rocha FAF, et al (2005) Horizontal cells in the retina of a diurnal rodent, the agouti (*Dasyprocta aguti*). Vis Neurosci 22:707–720. 16469182 10.1017/S0952523805226032

[45] Hughes A (1977) The topography of vision in mammals of contrasting life style: comparative optics and retinal organisation. In: Crescitelli F (Editor) Handbook of Sensory Physiology, Volume VII/5, The Visual System in Vertebrates, p. 613–756. Berlin, Germany: Springer-Verlag, 813 p.

[46] Stone J (1983) Parallel Processing in the Visual System. New York, New York, USA: Plenum Press, 438 p.

[47] Kremers J, Silveira LCL, Yamada ES, Lee BB (1999) The ecology and evolution of primate colour vision. In: Gegenfurtner KR, Sharpe LT (Editors) Color Vision: From Genes to Perception, p. 123–142. Cambridge, England, UK: Cambridge University Press, 492 p.

[48] Silveira LCL, Picanço-Diniz CW, Oswaldo-Cruz E (1989c) The distribution and size of ganglion cells in the retinae of large Amazon rodents. Vis Neurosci 2:221–235. 2562148 10.1017/s0952523800001140

[49] Silveira LCL (2004) Comparative study of the primate retina. In: Kaas JH, Collins CE (Editors) The Primate Visual System, p 29–51. Boca Raton, Florida, USA: CRC Press, 420 p.

[50] Collin SP (2012) The neuroecology of cartilaginous fishes: sensory strategies for survival. Brain Behav Evol 80:80–96. 22986825 10.1159/000339870

[51] Wikler KC, Rakic P (1996) Development of photoreceptor mosaics in the primate retina. Perspectives Dev Neurobiol 3:161–175. 8931091

[52] Williams AL, Reese BE, Jeffery G (2002) Role of retinal afferents in regulating growth and shape of the lateral geniculate nucleus. J Comp Neurol 445:269–277. 11920706 10.1002/cne.10171

[53] Finlay BL, Silveira LCL, Reichembach A (2005) Comparative aspects of visual system development. In: Kremers J (editor) The Primate Visual System: A Comparative Approach, p. 37–72. Chichester, England, UK: John Wiley & Sons, 367 p.

[54] Finlay BL (2008) The developing and evolving retina: using time to organize form. Brain Res 1192:5–16. 17692298 10.1016/j.brainres.2007.07.005

[55] Hendrickson AE (1994) Primate foveal development: a microcosm of current questions in neurobiology. Inv Ophthalmol Vis Sci 35:3129–3133. 8045707

[56] Springer AD, Hendrickson AE (2004) Development of the primate area of high acuity. 1. Use of finite element analysis models to identify mechanical variables affecting pit formation. Vis Neurosci 21:53–62. 15137581 10.1017/s0952523804041057

[57] Stone J, Johnston E (1981) The topography of primate retina: a study of the human, bushbaby, and new- and old-world monkeys. J Comp Neurol 196:205–223. 7217355 10.1002/cne.901960204

[58] Curcio CA, Allen KA (1990) Topography of ganglion cells in human retina. J Comp Neurol 300:5–25. 2229487 10.1002/cne.903000103

[59] Harman A, Abrahams B, Moore S, Hoskins R (2000) Neuronal density in the human retinal ganglion cell layer from 16–77 years. Anat Rec 260:124–131. 10993949 10.1002/1097-0185(20001001)260:2<124::AID-AR20>3.0.CO;2-D

[60] Perry VH, Cowey A (1988) The lengths of the fibres of Henle in the retina of macaque monkeys: implications for vision. Neuroscience 25:225–236. 3393279 10.1016/0306-4522(88)90021-8

[61] Wässle H, Grünert U, Röhrenbeck J, Boycott BB (1989) Cortical magnification factor and the ganglion cell density of the primate retina. Nature 341:643–646. 2797190 10.1038/341643a0

[62] Wässle H, Grünert U, Röhrenbeck J, Boycott BB (1990) Retinal ganglion cell density and cortical magnification factor in the primate. Vision Res 30:1897–1911. 2288097 10.1016/0042-6989(90)90166-i

[63] Herbin M, Boire D, Ptito M (1997) Size and distribution of retinal ganglion cells in the St. Kitts green monkey (*Cercopithecus aethiops sabeus*). J Comp Neurol 383:459–472. 9208993 10.1002/(sici)1096-9861(19970714)383:4<459::aid-cne5>3.0.co;2-1

[64] Fischer QS, Kirby MA (1991) Number and distribution of retinal ganglion cells in Anubis baboons (*Papio anubis*). Brain Behav Evol 37:189–203. 1878774 10.1159/000114358

[65] Wilder HD, Grünert U, Lee BB, Martin PR (1996) Topography of ganglion cells and photoreceptors in the retina of a New World monkey: the marmoset *Callithrix jacchus* . Vis Neurosci 13:335–352. 8737285 10.1017/s0952523800007586

[66] Webb SV, Kaas JH (1976) The sizes and distribution of ganglion cells in the retina of owl monkey, *Aotus trivirgatus* . Vision Res 16:1247–1254. 827113 10.1016/0042-6989(76)90049-3

[67] Hughes A (1971) Topographical relationships between the anatomy and physiology of the rabbit visual system. Doc Ophthalmol 30:33–159. 5000058 10.1007/BF00142518

[68] Coimbra JP, Hart NS, Collin SP, Manger PR (2013) Scene from above: retinal ganglion cell topography and spatial resolving power in the giraffe (*Giraffa camelopardalis*). J Comp Neurol 521:2042–2057. 23595815 10.1002/cne.23271

[69] Fleagle JG (1988) Primate Adaptation & Evolution. San Diego, California, USA: Academic Press, 486 p.

[70] Schein SJ (1988) Anatomy of macaque fovea and spatial densities of neurons in foveal representation. J Comp Neurol 269:479–505. 3372725 10.1002/cne.902690403

[71] Conradi N, Sjöstrand J (1993) A morphometric and stereologic analysis of ganglion cells of the central human retina. Graefe's Arch Clin Exp Ophthalmol 231:169–174. 8462891 10.1007/BF00920942

[72] Sjöstrand J, Conradi N, Klarén L (1994) How many ganglion cells are there to a foveal cone? A stereologic analysis of the quantitative relationship between cone and ganglion cells in one normal human fovea. Graefe's Arch Clin Exp Ophthalmol 232:432–437. 7926876 10.1007/BF00186586

[73] Sjöstrand J, Olsson V, Popovic Z, Conradi N (1999a) Quantitative estimations of foveal and extra-foveal retinal circuitry in humans. Vision Res 39:2987–2998. 10664798 10.1016/s0042-6989(99)00030-9

[74] Sjöstrand J, Popovic Z, Conradi N, Marshall J (1999b) Morphometric study of the displacement of retinal ganglion cells subserving cones within the human fovea. Graefe's Arch Clin Exp Ophthalmol 237:1014–1023. 10654171 10.1007/s004170050338

[75] Finlay BL, Darlington RB (1995) Linked regularities in the development and evolution of mammalian brains. Science 268:1578–1584. 7777856 10.1126/science.7777856

[76] LaVail MM, Rapaport DH, Rakic P (1991) Cytogenesis in the monkey retina. J Comp Neurol 309:86–114. 1894769 10.1002/cne.903090107

[77] Livesey FJ, Cepko CL (2001) Vertebrate neural cell-fate determination: lessons from the retina. Nature Rev Neurosci 2:109–118. 11252990 10.1038/35053522

[78] Dyer MA, Cepko CL (2001) Regulating proliferation during retinal development. Nature Rev Neurosci 2:333–342. 11331917 10.1038/35072555

[79] Hess RF, Hayes A, Field DJ (2003) Contour integration and cortical processing. J Physiol (Paris) 97:105–119. 14766137 10.1016/j.jphysparis.2003.09.013

[80] Doi E, Lewicki MS (2014) A simple model of optimal population coding for sensory systems. PLOS Comput Biol 10(8):e1003761 doi:10.1371/journal.pcbi.1003761 25121492 10.1371/journal.pcbi.1003761 PMC4133057

[81] Moran RJ, Symmonds M, Dolan RJ, Friston KJ (2014) The brain ages optimally to model its environment: evidence from sensory learning over the adult lifespan. PLOS Comput Biol 10(1):e1003422 doi:10.1371/journal.pcbi.1003422 24465195 10.1371/journal.pcbi.1003422 PMC3900375

[82] Troilo D, Howland HC, Judges SJ (1993) Visual optics and retinal cone topography in the common marmoset *Callithrix jacchus* . Vision Res 33:1301–1310. 8333154 10.1016/0042-6989(93)90038-x

[83] Bruesch SR, Arey LB (1942) The number of myelinated and unmyelinated fibers in the optic nerve of vertebrates. J Comp Neurol 77:631–665.

[84] Ogden TE, Miller RF (1966) Studies of the optic nerve of the rhesus monkey: nerve fibre spectrum and physiological properties. Vision Res 6:485–506. 4976686

[85] Potts AM, Hodges D, Shelman CB, Fritz KJ, Levy NS, Mangnall Y (1972) Morphology of the primate optic nerve. I. Method and total fiber count. Inv Ophthalmol Vis Sci 11:980–988. 4629435

[86] Rakic P, Riley KP (1983) Overproduction and elimination of retinal axons in the fetal rhesus monkey. Science 219:1441–1444. 6828871 10.1126/science.6828871

[87] Arey LB, Bickel WH (1935) The number of nerve fibers in the human optic nerve. Anat Rec 61 (Suppl.): 3.

[88] Oppel O (1967) Untersuchungen über Verteilung und Zahl der retinalen Ganglienzellen beim Menschen. Albrecht von Graefes Archiv für klinische und experimentelle Ophthalmologie 172:1–22. 5300622 10.1007/BF00577151

[89] Kupfer C, Chumbley L, Downer JC (1967) Quantitative histology of optic nerve, optic tract and lateral geniculate nucleus of man. J Anat 101:393–401. 6051727 PMC1270921

[90] Quigley HA, Addicks EM, Green WR (1982) Optic nerve damage in human glaucoma. III. Quantitative correlation of nerve fiber loss and visual field defect in glaucoma, ischemic neuropathy, papilledema, and toxic neuropathy. Arch Ophthalmol 100:135–146. 7055464 10.1001/archopht.1982.01030030137016

[91] Balazsi AG, Rootman J, Drance SM, Schulze M, Douglas GR (1984) The effect of age on the nerve fiber population of the human optic nerve. Am J Ophthalmol 97:760–766. 6731540 10.1016/0002-9394(84)90509-9

[92] Johnson BM, Miao M, Sadun AA (1987) Age-related decline of human optic nerve axon populations. Age 10:5–9.

[93] Repka MX, Quigley HA (1989) The effect of age on normal human optic nerve fiber number and diameter. Ophthalmology 96:26–31. 2919049 10.1016/s0161-6420(89)32928-9

[94] Jonas JB, Schmidt AM, Mullerbergh JA, Schlotzerschrehardt UM, Nauman GOH (1992) Human optic nerve fiber count and optic disc size. Inv Ophthalmol Vis Sci 33:2012–2018. 1582806

